# A Kinetic Study of CD83 Reveals an Upregulation and Higher Production of sCD83 in Lymphocytes from Pregnant Mice

**DOI:** 10.3389/fimmu.2017.00486

**Published:** 2017-04-26

**Authors:** Katrin Regina Helene Packhäuser, Gleyder Roman-Sosa, Jens Ehrhardt, Diana Krüger, Marek Zygmunt, Damián Oscar Muzzio

**Affiliations:** ^1^Research Laboratory, Department of Obstetrics and Gynecology, University of Greifswald, Greifswald, Germany; ^2^Département de Virologie, Unité de Virologie Structurale, Institut Pasteur, Paris, France

**Keywords:** CD83, pregnancy, tolerance, B cells, T cells

## Abstract

For the normal development of pregnancy, a balance between immune tolerance and defense is crucial. However, the mechanisms mediating such a balance are not fully understood. CD83 is a transmembrane protein whose expression has been linked to anti-inflammatory functions of T and B cells. The soluble form of CD83, released by cleavage of the membrane-bound protein, has strong anti-inflammatory properties and was successfully tested in different mouse models. It is assumed that this molecule contributes to the establishment of immune tolerance. Therefore, we postulated that the expression of CD83 is crucial for immune tolerance during pregnancy in mice. Here, we demonstrated that the membrane-bound form of CD83 was upregulated in T and B cells during allogeneic murine pregnancies. An upregulation was also evident in the main splenic B cell subtypes: marginal zone, follicular zone, and transitional B cells. We also showed that there was an augmentation in the number of CD83^+^ cells toward the end of pregnancy within splenic B and CD4^+^ T cells, while CD83^+^ dendritic cells were reduced in spleen and inguinal lymph nodes of pregnant mice. Additionally, B lymphocytes in late-pregnancy presented a markedly higher sensitivity to LPS in terms of CD83 expression and sCD83 release. Progesterone induced a dosis-dependent upregulation of CD83 on T cells. Our data suggest that the regulation of CD83 expression represents a novel pathway of fetal tolerance and protection against inflammatory threats during pregnancy.

## Introduction

Pregnancy is associated with several immune adaptations that allow the growth of a healthy semi-allogeneic fetus. These include a finely regulated shift from a Th1 and Th17 toward a Th2 tolerogenic response driven by anti-inflammatory molecules secreted by, among others, the “so-called” regulatory cells (1). Several cell types have been described with regulatory functions during pregnancy, including T (2) and B lymphocytes (3). Lymphocytes with regulatory properties allow the eradication of external threats and limit proinflammatory responses. In particular, the presence of regulatory cells at the maternal–fetal interface is crucial in mammals with invasive placentation (4). A potential dysregulation of regulatory cells may have several consequences on the human health and has been associated with autoimmune diseases, cancer, chronic infections (5, 6), and pregnancy-related disorders, including preterm birth, preeclampsia, and recurrent pregnancy loss (7–10). CD4^+^CD25^+^ regulatory T cells and CD19^+^CD24^hi^CD27^+^ regulatory B cells are increased during normal pregnancies, while some cases of pregnancy loss associate with a lack of this increment (8, 11). In mouse models for pregnancy disturbances, the transfer of regulatory lymphocytes rescues mice from normally occurring pregnancy loss (12, 13).

An increasing body of evidence links inflammation in the absence of infection to the onset of preterm labor (14–16). For this reason, inflammatory pathways emerged as therapeutic targets to handle certain cases of preterm labor (17–19). Immune cells apply several strategies to exert their immunomodulatory functions. These include the release of factors, such as cytokines and chemokines, and expression of membrane ligands, including suppressive ligands (LFA-1, CTLA-4) and apoptosis-related ligands (PD-L1, TRAIL) (20–23). To this group belongs the CD83, which is a transmembrane molecule of the immunoglobulin family, first described as an activation marker for dendritic cells (DCs) (24, 25).

In DCs, CD83 is mostly associated to the maturation state and functions as a costimulatory molecule for T cell activation, independently of the level of expression (26–29). By targeting activated DCs, anti-CD83 depleting antibodies can prevent human peripheral blood mononuclear cell-induced acute graft vs. host disease in SCID mouse recipients (30, 31). Although the mechanisms through which membrane-bound CD83 regulates immune responses are still to be elucidated, one of the proposed pathways is though negative regulation of MARCH1 and MARCH8. These E3 ubiquitin-protein ligases control the ubiquitination of MHCII molecules, impacting on their turnover on DCs and B cells (32, 33). On the other hand, mice with CD83^−/−^ DCs develop exacerbated dextran sodium sulfate induced colitis (34). CD83 on DCs can also regulate cytokine production of immature DCs through cell–cell contact, resulting in reduced secretion of MCP-1 and IL-12p40 (34).

CD83 expression can be easily detected on activated T cells (35–37). While reports implicating silencing of CD83 expression on antigen-specific CD4^+^ T cells showed an impaired proliferative capacity and reduced cytokine secretion, this may not apply to all CD4^+^ T cell populations or it may represent a mechanism to avoid over-reactive CD4^+^ T cells (38–40). Indeed, CD83 overexpression in murine naïve CD4^+^ T cells induces regulatory T cell-specific FOXP3 expression and confers *in vivo* tolerogenic properties (39).

Studies focused on B cells also show a correlation of CD83 and B cell activation (41). CD83 co-localizes with the BCR as well as with the LPS receptor and regulates signal transduction pathways downstream both receptors (42). On BCR, CD83 acts reducing its sensitivity, for which it may prevent over-reaction of activated B cells. BCR sensitivity is also thought to be a determinant for the MZ vs. FO B cell developmental decision (43). This may explain the MZ over FO B cell preference in the maturation of CD83 over-expressing B cells mice (44). When B cells are transferred with CD83, LPS stimulation leads to high IL-10 production derived from MZ B cells. CD83 expression on B cells also predisposes FO B cells to cell death. CD83-expressing B cells also respond with reduced IgG production. Taken together, CD83 expression on B cells is associated to a MZ over a FO B cell preference and anti-inflammatory B cell responses. As well as in mice with CD83 over-expressing B cells, normal pregnancies display several B cell adaptations that include a preference for MZ over FO maturation and a higher B cell-derived IL-10 production (12, 45, 46). This phenomenon seems to be reflected in the antibody profile of pregnant mice by elevated MZ B cell-derived Ig types IgM, IgA, and IgG3 (43).

Beyond the influence of membrane CD83 on lymphocyte function, an anti-inflammatory role of soluble CD83 was depicted in different models of autoimmune diseases as well as allograft transplantation (47–53). Soluble CD83 is very likely generated either by shedding of the membrane-bound protein (54–56) or by alternative splicing of the transcript (57). Due to the growing line of evidence of the therapeutic potential of exogenous sCD83 in inflammatory and autoimmune diseases, we hypothesize that it can be a tolerogenic molecule that might support pregnancy as well.

In this work, we show that the expression of the membrane molecule CD83 as well as its soluble form, sCD83, is increased in the course of normal murine pregnancies. These results suggest that sCD83 may play a role in the maintenance of pregnancy.

## Materials and Methods

PBS, FBS, PenStrep, PMA, and RPMI1640 were purchased from Merck Millipore (Billerica, MA, USA) and estrogen, progesterone, and LPS from Sigma-Aldrich Chemie GmbH (Munich, Germany). Following antibodies were purchased from BD Bioscience (Heidelberg, Germany): CD83 (Michel-19); B220 (RA3-6B2); CD4 (RM4-4 and RM4-5); CD23 (B3B4); CD21 (7G6); CD19 (1D3); CD11c (HC3); CD25 (PC61); CD69 (H1.2F3); TNF (MP6-XT22); IFN (XMG1.2); IL-17 (TC11-18H10); FoxP3 (R16-715); IL-10 (JES5-16E3); and Ki-67 (B56), as well as Purified NA/LE CD3e (145-2C11). DC Marker DCIR2 (33D1), Brefeldin A, and anti-mouse CD83 purified antibody were purchased from eBioscience (San Diego, CA, USA) and isotype-control (Purified Rat IgG1k isotype) from BioLegend (San Diego, CA, USA).

### Animals

C57Bl6/J female mice and BALB/c male mice were purchased from Charles River (Sulzfeld, Babavia, Germany) or Janvier Labs (Saint-Berthevin Cedex, France). BALB/c males were bred in our Central Service and Research Facility for Animals (ZSFV). The animals were kept co-housed in a 12L:12D cycle with food and water *ad libitum*. Eight- to twelve-week-old C57Bl6/J female mice were paired with BALB/c males and checked for vaginal plug every morning. Observation of plug was declared day 0 of pregnancy, and the female was separated from the male. Mice were sacrificed at day 7, 14, or 18 post plug (7, 14, and 18 dpp); spleen, paraaortic lymph nodes (PLN), inguinal lymph nodes (ILN), and thymus as well as serum were collected. We performed permanent matings and mice representative of all stages of pregnancy were available weekly. Non-pregnant C57Bl6/J female mice were randomly sacrificed used as control.

### Cell Preparation

Single cell suspensions from paraaortic lymph nodes, inguinal lymph nodes, and thymus were obtained. The tissue was carefully squeezed through a 40-µm nylon cell strainer and washed with PBS. In case of spleen tissue, an erythrocyte lysis with 10 mL Lysis Buffer (0.89% NH4Cl, 0.1% KHCO3, 0.003% 0.5 M EDTA) was performed and stopped after 5 min with 3 mL FBS. After washing, the cell suspension was filtered a second time with a 40-μm cell strainer. The cell counts of the suspensions were determined using a Neubauer chamber.

### Flow Cytometry

Flow cytometry was applied to evaluate the expression of CD83 on B, T, or DCs from spleen, thymus, and lymph nodes. Cell suspensions were first incubated with CD16/32 mAb Fc block (BD Pharmingen, Heidelberg, Germany) for 5 min. Staining with fluorochrome-labeled specific antibodies was performed for 30 min at 4°C in the dark. Samples from spleen were additionally stained with Fixable Viability Dye eFluor 780 (eBioscience, San Diego, CA, USA) for 30 min and later washed with FACS buffer (1% BSA, 0.1% NaN3, 0.955% PBS) before Fc blocking. Data were acquired on FACSCanto (BD Bioscience) and analyzed by using FlowJo software (FlowJo, LLC, Ashland, TN, USA). Information about gating strategies can be provided upon request.

### Cell Stimulation

The 1 × 10^6^ splenic lymphocytes were cultured 48 h in a total volume of 500 µL RPMI 1,640 culture medium supplemented with 10% FBS and antibiotics on 48-well flat-bottom suspension plates. The stimulation was performed with either 10 µg/mL LPS for 48 h and 50 ng/mL PMA as well as 500 ng/mL ionomycin for the last 5 h. In order to examine the influence of pregnancy related hormones on CD83 expression, the cells were stimulated with different concentrations of estrogen (5 pg/mL, 100 pg/mL, and 100 ng/mL) or progesterone (50 ng/mL; 500 ng/mL; 5,000 ng/mL;50,000 ng/mL). After stimulation cells and supernatants were separated by 5 min centrifugation at 1,300 × *g*. The supernatants were stored at −80°C, and the cells were directly used for Flow Cytometry.

### Cell Depletion

To determine which cells produce soluble CD83, splenic lymphocytes were depleted from CD4^+^ or CD19^+^ cells. CD19 depletion was performed using CD19 MicroBeads mouse (Miltenyi Biotec GmbH, Teterow, Germany). For CD4 depletion, a negative selection Mouse CD4^+^ T-Cell Isolation kit was purchased from EasySep (STEMCELL Technologies, Vancouver, BC, Canada). In both cases, the supplier’s instructions were followed; 500,000 cells were cultured with 250 µL culture medium and stimulated with LPS as described before.

### ELISA

The levels of sCD83 in sera and supernatants were measured using an ELISA kit for CD83 (Cloud-Clone Corp., Houston, TX, USA). The serum samples were diluted 1:2 in PBS, while the supernatants were examined without dilution. The test was performed following the instruction manual.

### Real-time PCR

Magnetic isolated splenic CD19^+^ B cells were treated with TriFast peqGOLD (VWR, Radnor, PA, USA). RNA isolation, cDNA synthesis, and real-time PCR were performed as previously described (58). RNA concentration was evaluated spectrophotometrically using the NanoPhotometer PEARL (IMPLEN, Munich, Germany). Samples were amplified in duplicate and nontemplate samples were used as controls. Primer pairs were chosen to span an exon–exon junction and so avoid unwanted genomic DNA amplification. Real-time PCR was performed using SYBR Green (AB/Life Technologies, Darmstadt, Germany) in a 7300 Real-time PCR System (Applied Biosystems, Darmstadt, Germany) with β-actin as housekeeping gene. Primer sequences were the following: CD83 Fw: TGAAGGTGACAGGATGCCC; CD83 Rw: CTTGGGGAGGTGACTGGAAG; β-actin Fw: TGGAATCCTGTGGCATCCATGAAAC; Rw: TAAAACGCAGCTCAGTAACAGTCC. All primers were purchased from Invitrogen (Carlsbad, CA, USA).

### Recombinant sCD83 Production

#### Cloning of the DNA Encoding for the Murine CD83 Ectodomain

pGRS-88: The gene fragment was amplified by PCR with the primers O-GRS-112 (aattatt*TCTAGA*GCCACCATGTCGCAAGGCCTCCAGCTCCTGTTTC) and O-GRS-113 (GCTCCTGTACTTCCTGAAAGTTGACTCTGTAG) with the Phusion polymerase (NEB, Ipswich, MA, USA) from RNA isolated from stimulated lymphocytes. The PCR product was digested with *Xba*I and phosphorylated with T4 polynucleotide kinase 3′ phosphatase minus (NEB, Ipswich, MA, USA) and ligated to the vector pGRS-12 (59) digested with *Xba*I (NEB, Ipswich, MA, USA) and *Afe*I (NEB, Ipswich, MA, USA).

#### Expression and Purification of the Murine CD83 Ectodomain

The CD83 ectodomain was purified from supernatant of transiently transfected eukaryotic cells essentially a previously described (59). In brief, HEK-293T cells transfected with the DNA mixed with branched polyethylenimine (PEI) (Sigma-Aldrich Chemie GmbH, Munich, Germany). Three days after transfection, the supernatant was collected and concentrated (Vivaflow system, cassette with membrane of MW CO 5,000 Da; Sartorius Stedim, Göttingen, Germany). The expression of sCD83 on transfected cells was controlled by immune fluorescence using anti-CD83 antibody. sCD83 functionality was checked by its ability to reduce the generation of CD80^+^ bone marrow-derived DCs (60). We obtained a reduction of almost 50% of CD80^+^ cells. Then the protein was purified using streptactin superflow high capacity slurry (IBA Biotech, Göttingen, Germany) according to the instructions of the manufacturer. The presence of the protein in each collected fraction was checked by SDS-PAGE (61) and staining with Instant Blue (Expedeon, San Diego, CA, USA). For all our experiments, we used sCD83 purified from the same supernatant.

### sCD83 Effect on T Cells

The 96-Well plates were coated with CD3e (1 µg/mL) and incubated overnight at 4°C. T cells from murine lymph nodes were isolated using a negative selection Mouse CD4^+^ T Cell Isolation kit (STEMCELL Technologies, Vancouver, BC, Canada). A total of 100,000 cells were incubated overnight in 100 µL supernatants (see Cell Stimulation) with 10% FBS. To evaluate an effect of sCD83, either an Anti-Mouse CD83 Purified Antibody or an Isotype-Control were added.

Additionally, T Cells were incubated overnight in 100 µL culture medium and stimulated with a recombinant sCD83 protein (2 and 200 ng/mL, “CD83-low” and “CD83-high,” respectively). As control, the amino terminal domain of the Schmallenberg glycoprotein Gc, which was produced and isolated in the same manner (59), was used in equivalent concentrations. LPS, PMA, and ionomycin were added as described before.

For intracellular staining, Brefeldin A was added for the last 5 h. Cells were collected and stained with fixable viability dye eFluor 660 and later with specific membrane antibodies. After extracellular staining, cells were fixated with fixation/permeabilization kit (eBioscience, San Diego, CA, USA), for 20 min, permeabilized using permeabilization buffer (eBioscience, San Diego, CA, USA), and intracellular stained with specific antibodies for 30 min.

### Statistical Analysis

Normality was assessed by D’Agostino and Pearson omnibus normality test. MFI-data, serum sCD83 ELISA, and PCR of stimulated lymphocytes were analyzed using Kruskal–Wallis test with Dunn’s posttest. Hormones stimulations experiments as well as lymphocyte depletion test was analyzed by ANOVA and Dunnett’s test. *In vitro* secretion of sCD83 by ELISA in stimulated and nonstimulated np and 18 dpp isolated splenocytes was analyzed using two-way-ANOVA with Bonferroni posttests. Further data were analyzed by ANOVA with Tuckey’s posttest. Significant differences between groups were indicated with asterisks as follows: *p < 0.05; **p < 0.01; and ***p < 0.001.

## Results

### CD83 Expression Is Upregulated in B and T Lymphocytes but Not in DCs during Pregnancy

**Figure 1:**
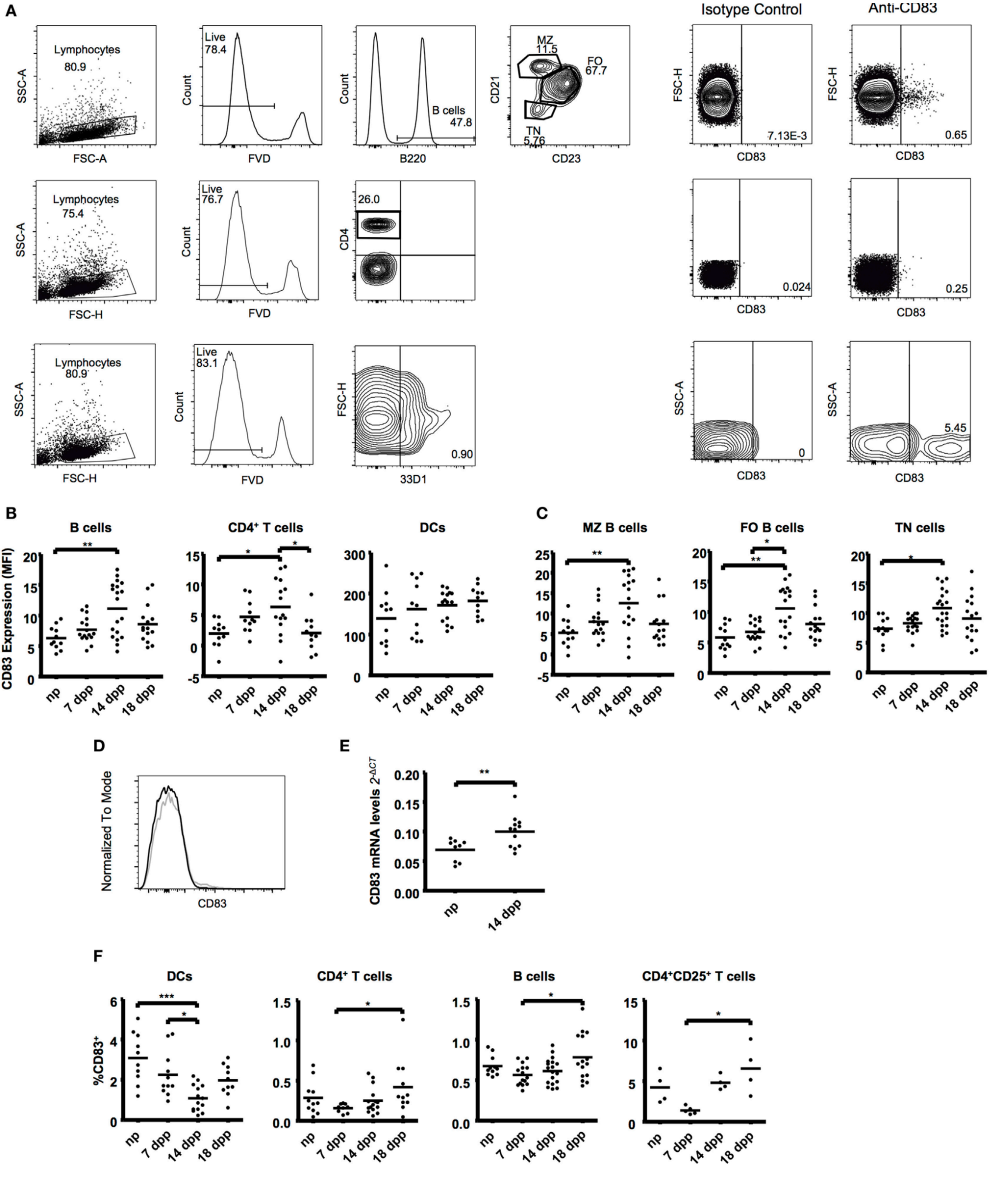
**B cells (B220^+^), T cells (CD4^+^), and dendritic cells (33D1^+^) from fresh spleen tissue were examined in the course of normal murine pregnancies**. **(A)** Representative plots showing gating strategies to CD83 expression (right). **(B)** Scatter dot plots show the median fluorescence intensity of CD83 within the respective cell types in live lymphocytes. **(C)** B220^+^ B cells were further subdivided in CD21/35^hi^CD23^low^ marginal zone B cells (MZ), CD21/35^int^CD23^hi^ follicular zone B cells (FO), and CD21/35^−^CD23^−^ transitional B cells (TN). Scatter dot plots show the mean fluorescence intensity of CD83. **(D)** Representative histogram overlapping CD83 expression on B cells of a non-pregnant mice (black line) or pregnant mice at day 14 (gray line). **(E)** Magnetic isolated splenic CD19^+^ B cells were analyzed for mRNA expression levels of CD83 by quantitative real-time PCR. FACS data were analyzed by Kruskal–Wallis test with Dunn’s posttest. PCR was analyzed by Student’s *t*-test. Significant differences are indicated (*p ≤ 0.05, **p ≤ 0.01). **(F)** Data show the percentage of CD83^+^ cells within the respective cell types in spleen. T cells were further characterized within the CD25^+^ population. Data show the percentage of CD83^+^ cells within CD25^+^ T cells. Data were analyzed by ANOVA with Tuckey’s posttest. Significant differences are indicated (*p ≤ 0.05, ***p ≤ 0.001).

In order to characterize the dynamics of CD83 during the course of normal murine pregnancy, we analyzed CD83 expression at different time points of normal murine pregnancies. We evaluated the presence of CD83 on CD11c^+^ and DCIR2^+^ dendritic cells, CD4^+^ T cells, and different B-cell populations (Figure 1A).

In spleen, we found an upregulation of CD83 not only on B cells but also on CD4^+^ T cells at day 14 of pregnancy (1.76- and 3.18-fold increase, respectively, Figures 1B,C). A deeper analysis within the major splenic B cell populations—transitional (TN), marginal zone (MZ), and follicular zone (FO) B cells—showed that the CD83 upregulation at day 14 also occurs in all subsets (1.47-, 2.76-, and 1.80-fold increase, respectively, Figures 1C,D). This increase was also confirmed by analyzing mRNA levels of CD83 on fresh-isolated B cells (Figure 1E).

**Figure 2:**
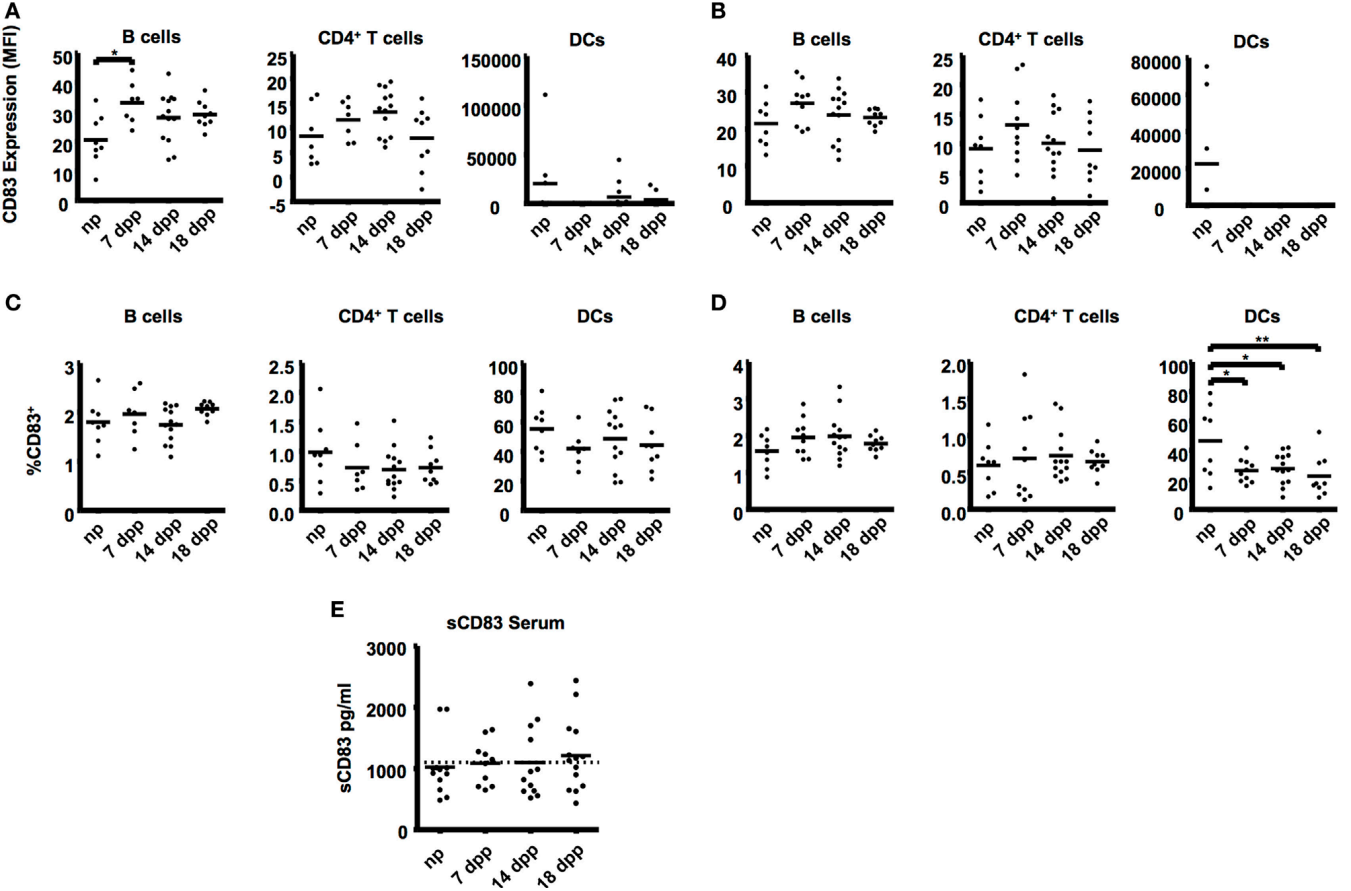
**B cells (B220^+^), T cells (CD4^+^), and dendritic cells (33D1^+^) from uterine-draining lymph nodes were examined in the course of pregnancy**. **(A)** Scatter dot plots show the mean fluorescence intensity of CD83 within the respective cell types in paraaortic lymph nodes. **(B)** Scatter dot plots show the mean fluorescence intensity of CD83 within the respective cell types in inguinal lymph nodes. Data were analyzed by Kruskal–Wallis test with Dunn’s posttest. Significant differences are indicated (*p ≤ 0.05). **(C)** Percentage of CD83^+^ cells within the respective cell types in paraaortic lymph nodes **(C)** and inguinal lymph nodes **(D)**. Data were analyzed by ANOVA with Tuckey’s posttest. Significant differences are indicated (*p ≤ 0.05, **p ≤ 0.01). **(E)** Sera of pregnant and non-pregnant mice were analyzed by ELISA to determine sCD83 levels during pregnancy. Data were analyzed by Kruskal–Wallis test with Dunn’s posttest.

The analysis of the percentages of CD83^+^ cells depicted a significant increase of positive B and T cells (1.36- and 2.65-fold increase in B and CD4^+^ T cells, respectively, Figure 1F) from early (7 dpp) to the end of pregnancy (18 dpp). DCs, however, showed a reduction of the percentage of CD83^+^ cells during pregnancy, being statistically significant at day 14 of pregnancy (3.08 ± 0.40 in np vs. 1.08 ± 0.17 in 14 dpp, Figure 2A). After an in-depth analysis of T cell subsets, an increase in the percentage of CD83^+^ cells among regulatory CD4^+^CD25^+^ cells was observed (from 1.39 ± 0.21 at early pregnancy to 6.54 ± 1.52 at advanced pregnancy, Figure 1F).

An upregulation of CD83 was also found in B cells from the uterus-draining paraaortic lymph nodes (MFI: 33.10 ± 2.68 in 7 dpp vs. 20.54 ± 2.98 in np, Figure 2A). In ILN, though a similar tendency was observed, no significant differences were found among the groups (Figure 2B). Neither B- nor CD4^+^ T cells showed differences in the percentages of CD83^+^ cells in PLN or ILN (Figures 2C,D). Significantly lower percentages of CD83^+^ in DCs were also observed on ILN of all groups of pregnant mice (55.23 ± 5.58, 41.86 ± 4.40, 48.7 ± 5.58, 44.28 ± 5.72 in np, 7, 14, and 18 dpp respectively, Figure 2B). No significant differences were found on either the CD83 expression or the percentage of CD83^+^ cells in any cell type in thymus (data not shown).

*In vivo* levels of sCD83 were determined from serum samples (Figure 2E). No statistically significant changes in the mean levels of sCD83 were seen. Nevertheless, only 16.7% of the non-pregnant mice had sCD83 serum levels over 1,100 pg/mL (the mean value for all groups of mice), while this percentage was higher in pregnant mice (50.0% in mice at 7 dpp, 25.0% at 14 dpp, and 57.7% at 18 dpp).

### Progesterone Induces Higher CD83 Expression

**Figure 3:**
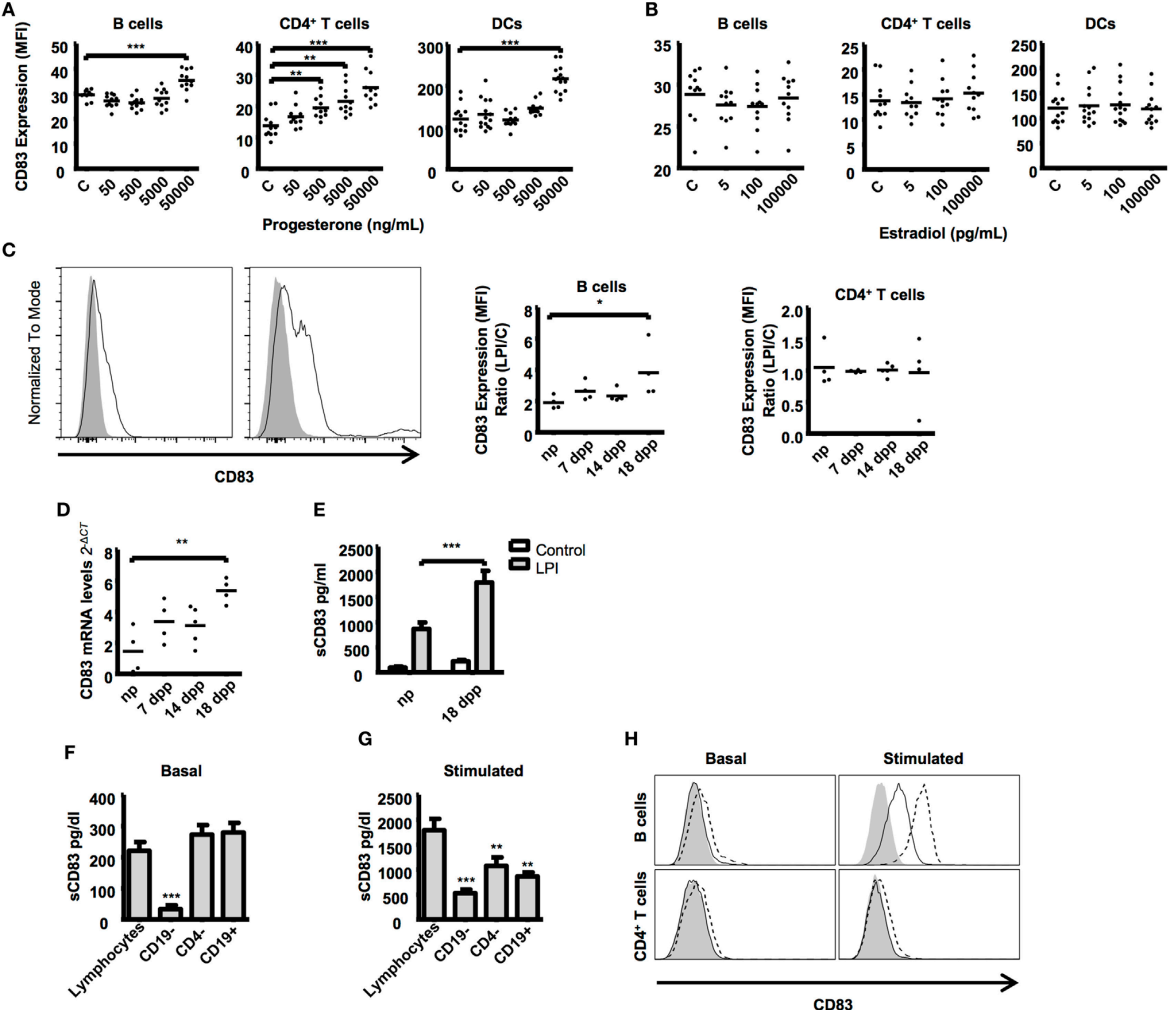
**Splenic lymphocytes from different stages of murine pregnancy were treated with key pregnancy hormones, progesterone and estrogen in increasing concentrations for 48 h**. Cells cultured in medium alone served as control. Scatter dot plots show the CD83 expression on CD19^+^ B cells, CD4^+^ T cells. and CD11c^+^ dendritic cells after progesterone **(A)** or estrogen **(B)** stimulation. Data were analyzed by Kruskal–Wallis test with Dunn’s posttest. Significant differences are indicated (**p ≤ 0.01, ***p ≤ 0.001). **(C)** Splenic lymphocytes from different stages of murine pregnancy were stimulated with LPS for 48 h plus PMA and ionomycin for the last 5 h. The histogram shows a representative stimulation from a non-pregnant mouse B cells (left) and a pregnant mouse at day 18 (right). Gray curves show basal CD83 expression, the black line represents CD83 expression after stimulation. Scatter dot plots show the quotient between CD83 MFI of LPS-stimulated and nonstimulated CD19^+^ B cells (left) and CD4^+^ T cells (right). **(D)** mRNA expression levels of CD83 by quantitative real-time PCR of stimulated lymphocytes relative to nonstimulated controls. **(E)** Bars compare the amount of sCD83 in the supernatant from LPS-stimulated lymphocytes from non-pregnant and pregnant mice from late-pregnancy stage and nonstimulated controls. Data were analyzed by two-way-ANOVA with Bonferroni posttests **(C)**. Significant differences are indicated (*p ≤ 0.05, **p ≤ 0.01, ***p ≤ 0.001). **(F)** CD19- or CD4-depleted splenic lymphocytes and magnetic isolated splenic CD19^+^ B cells from mice at advanced pregnancies were cultured for 48 h. Data show the basal production of sCD83 in the supernatants in nonstimulated lymphocytes **(F)** or after stimulation with LPS, PMA, and ionomycin **(G)**. **(H)** Overlapping histogram display differences between nonstimulated (left) and stimulated (right) B cells (top) and CD4^+^ T cells (bottom). Filled curved represents the FMO for CD83, while filled and dashed lines show extracellular and intracellular CD83 staining, respectively. Data were analyzed with ANOVA and Dunnett’s test to compare treatments against non-depleted controls. Significant differences are indicated (*p ≤ 0.05, **p ≤ 0.01, ***p ≤ 0.001).

Sexual hormones estradiol and progesterone influence a number of immune and non-immune adaptations to pregnancy (62–64). We observed that CD83 expression during pregnancy had a similar pattern to progesterone levels, with a maximum at day 14 of pregnancy (65). To test if progesterone or estradiol influences CD83 expression, we isolated splenocytes and stimulated them with increasing concentrations of estradiol and progesterone. Progesterone but not estradiol upregulated CD83 expression on B cells and DCs (Figure 3A). T cells, on the other hand, reacted to progesterone stimulation more sensitively and upregulated CD83 expression in a dose-dependent manner (Figure 3A). Estrogen stimulation failed to induce changes in CD83 expression also on T cells (Figure 3B).

### Lymphocytes from Advanced Pregnant Mice Release More sCD83

The upregulation of CD83 both in B and T cells (Figure 1B) and in response to progesterone (Figure 3A) prompted us to assess the expression pattern of cell surface and soluble CD83 in lymphocytes of pregnant mice upon stimulatory challenge. We found that B cells from advanced pregnancy express much higher levels of CD83 when stimulated with LPS, PMA, and ionomycin (a combination we shortened as LPI) (3.82 ± 0.85-fold increase in 18 dpp vs. 1.91 ± 0.21-fold increase in np, Figure 3C). We did not observe any significant effect on the T cells population we analyzed (Figure 3C). CD83 mRNA levels were also analyzed in LPI-stimulated B cells. The B cells from mice with advanced pregnancies displayed an almost fourfold increase in the levels of CD83 mRNA when compared with the cells from np mice (Figure 3D).

Taking into account the *in vivo* levels of sCD83 observed in sera of pregnant vs. non-pregnant mice, we sought to better characterize sCD83 production. We used the supernatant of the cultured cells to assess sCD83 production by ELISA. In agreement with CD83 MFI and mRNA levels on B cells, we found that sCD83 was present in significantly higher concentrations in the supernatant of stimulated lymphocytes of advanced pregnant mice compared with the supernatant of lymphocytes of non-pregnant mice (1,793 ± 226.1 pg/mL in 18 dpp vs. 868.4 ± 124.9 pg/mL in np, Figure 3E). In summary, lymphocytes of late-pregnancy mice upregulate the expression of membrane CD83 and release more sCD83 upon stimulation than lymphocytes of non-pregnant mice do.

### B Cells Are the Main Source of sCD83

Several lymphocyte populations present in the spleen express CD83 and have the potential to release sCD83. To identify the main source of splenic sCD83, we isolated splenocytes and further depleted CD19^+^ B and CD4^+^ T cells. Depleted splenocytes were stimulated to produce sCD83 as in Figure 3C, and levels of sCD83 in the supernatant of the cell culture were assessed by ELISA. Remarkably, CD19^+^ B cell depletion had the strongest negative effect on the basal release of sCD83 (220.4 ± 28.29 pg/mL in total splenocytes vs. 28.48 ± 14.3 in CD19 depleted splenocytes, Figure 3F) as well as in the LPI induced release (1,793 ± 226.1 pg/mL in total splenocytes vs. 527.0 ± 75.52 pg/mL in CD19 depleted splenocytes, Figure 3G). Remarkably, there is no significant difference between purified CD19^+^ B cells release of sCD83 and total splenocytes (220.4 ± 28.29 pg/mL in total splenocytes vs. 279.4 ± 68.29 pg/mL in CD19^+^ B cells). However, upon stimulation, depletion of CD4^+^ T cells had as well a slightly detrimental effect (1,793 ± 226.1 pg/mL in total splenocytes vs. 1,077 ± 169.3 pg/mL in CD4 depleted splenocytes, Figure 3G); purified CD19^+^ B cells release of sCD83 is lower than that in total splenocytes (1,793 ± 452.2 pg/mL in total lymphocytes vs. 864.34 ± 176.8 pg/mL in CD19^+^ B cells), indicating that T cells might participate on the B cell activation for the release of sCD83. We also observed that under LPI stimulation, B cells produced display higher intracellular CD83 staining than do T cells under same conditions (Figure 3H). To sum up, B cells represent the main source of splenic sCD83 release upon LPI stimulation.

### sCD83 Influence on T Cell Activity *In Vitro*

**Figure 4:**
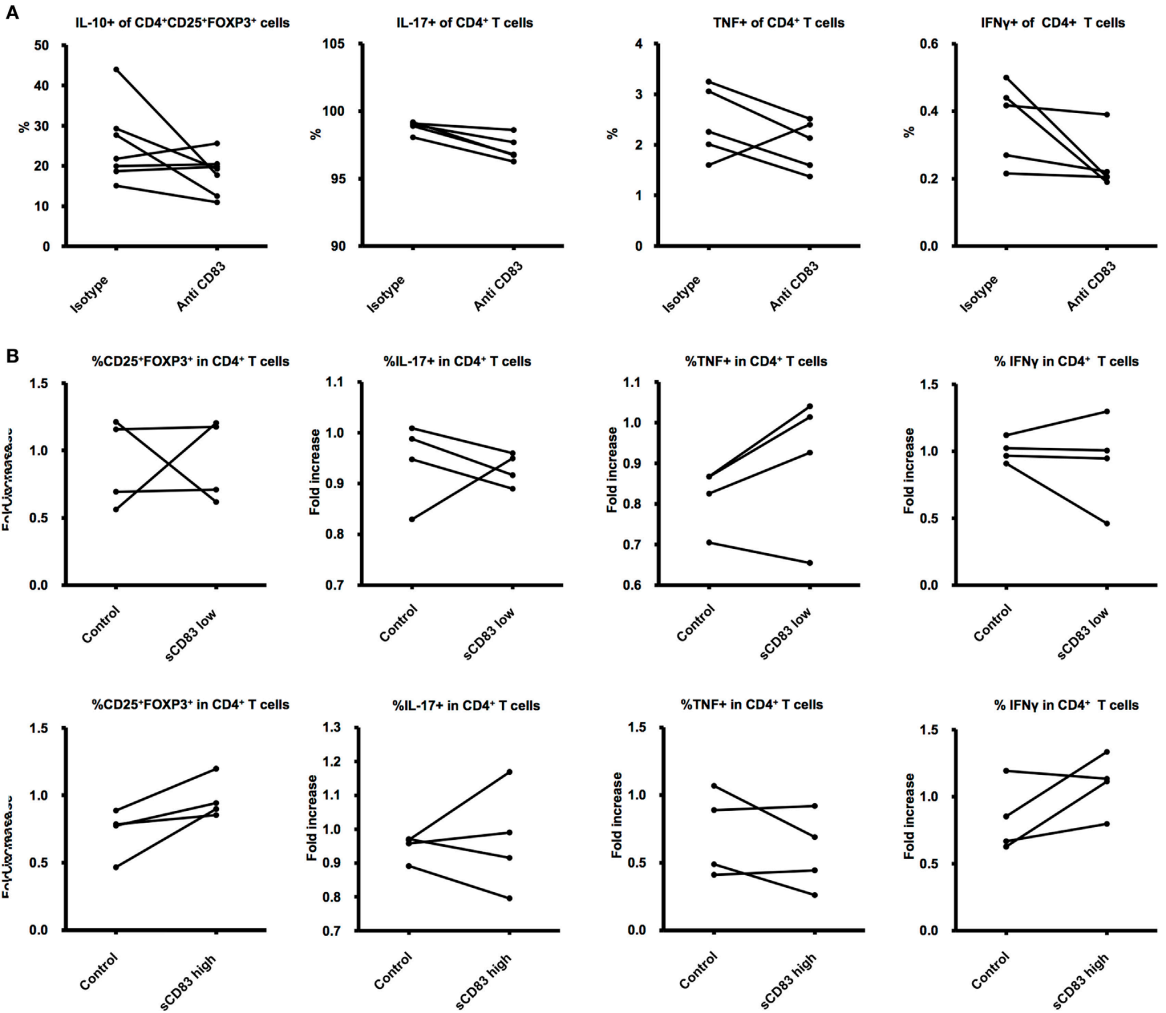
**Effects of sCD83 on T cells**. **(A)** CD4^+^ T cells were isolated from lymph nodes and cultured 24 h with supernatant obtained from the experiments on (Figure 1C) and treated with a blocking antibody against CD83 or an isotype control. **(B)** CD4^+^ T cells were isolated from lymph nodes and cultured 24 h with LPS, PMA, ionomycin, and either recombinant sCD83 or a control protein in similar concentrations. The cells were later analyzed for expression of activation markers and associated intracellular and surface markers. Student’s *t*-test was used to compare differences between the groups.

The soluble form of CD83 has been shown to possess *in vivo* as well as *in vitro* anti-inflammatory properties (47–53, 57). Taking into account the presence of sCD83 in the supernatants of stimulated splenocytes, we used the supernatant to stimulated naïve T Cells. In some experiments, we attempted to block sCD83 with a specific antibody against CD83. The presence of a blocking antibody in the cell culture failed to significantly reduce the cytokine expression of CD4^+^ T cells (Figure 4A).

sCD83 was previously shown to affect T-cell function via DCs, leading to higher percentages of CD4^+^CD25^+^Foxp3^+^ Tregs, lower production of proinflammatory cytokines, and increasing the release of immunosuppressive cytokines (47), all features that promote pregnancy wellbeing (2). To test the direct role of sCD83 on naïve CD4^+^ T cells, we generated recombinant sCD83 using methods already described (59). The recombinant protein was used to treat isolated CD4^+^ T cells. After 48 h of direct stimulation, we were not able to show a direct effect of sCD83 on T cells (Figure 4B).

## Discussion

The fact that the immune system can mount a response to pathogen threats without compromising the health of a semi-allogeneic fetus has been a matter of major interest among reproductive biologists. We wondered if CD83 expression was one of the regulators of immune responses that accompany normal pregnancy.

In the case of B lymphocytes, CD83 overexpression induces an anti-inflammatory phenotype, by reduction of Ig secretion and induction of IL-10 secretion (66). Our data show an upregulation of membrane-bound CD83 on splenic and paraaortic lymph nodes B cells, which correlates with the increase of IL-10 producing B cells observed during healthy murine (12) and human pregnancies (8). We also found that B cells are especially sensitive at the end of pregnancy to inflammation triggers (PMA, LPS) in terms of CD83 upregulation and sCD83 secretion. Our data suggest that CD83 expression may be a key regulator which shapes B-cell function into a beneficial phenotype for late-pregnancy events, such as inflammation-triggered preterm birth (10).

We also found that B cells were the main source of the basal sCD83 release in the supernatant of cultured splenocytes. However, after stimulation, CD4^+^ T cell depletion has a detrimental effect as well, indicating that T cells may cooperate in B-cell activation for subsequent sCD83 release. Kretschmer et al. previously showed that activated T cells support the CD83 upregulation on B cells via CD40-CD40L interactions (67).

Studies addressing CD83 expression on T cells have led to controversial results, which vary from effects on the stimulation of naïve and memory T cells (68), on the production of proinflammatory cytokines (38), or even correlating with immunosuppressive functions (39, 69). In our experiment, we showed that CD83 is upregulated on CD4^+^ T cells during pregnancy and the proportion of CD4^+^CD25^+^ T cells that are positive for CD83 increases throughout the stages of pregnancy. CD83 expression particularly on CD4^+^CD25^+^ T cells is linked to immunosuppressive phenotype (39, 40). Additionally, our results are in agreement with the well-studied pregnancy-supporting role of regulatory T cells (70, 71).

Many of the changes we observed concerning CD83 expression on lymphocytes were significant from mid-pregnancy, when estradiol and progesterone are increasing in mice as well as in human pregnancies (65, 72). B as well as T lymphocytes express estrogen and progesterone receptors (73–76). Regulatory T cells, as well as other immune cells, are regulated by sex hormones during pregnancy (77, 78). In our experiments, we showed that progesterone had a dose-dependent effect on CD83 expression on CD4^+^ T cells. We also observed progesterone-specific effects on CD83 expression on B Cells, but only by reaching high concentrations of the hormone in cell culture. When we used similar concentrations to serum levels observed in murine pregnancies, we were not able to show any significant differences in the CD83 expression on lymphocytes. We cannot exclude that progesterone may be involved in the changes observed on B cell CD83 expression during the course of pregnancy, since a relatively short stimulation was performed. On the other hand, our results support anti-inflammatory therapeutic use of progesterone that is based on high doses of the hormone to prevent preterm labor (79).

Having demonstrated the higher capacity of B lymphocytes from pregnant mice to release sCD83, we decided to test a direct effect of sCD83 on CD4^+^ T cells *in vitro*. The mechanism of action of sCD83 has not been unveiled yet, but it has been proposed that may act through blocking of the natural ligand of CD83 (48). In our experiment involving the supernatant of stimulated lymphocytes, the blocking antibody that remained in the culture media may be altering the CD83/CD83-ligand interaction, leading to an apparent less T cell activation. In our attempt to block sCD83 to evidence more CD4^+^ activation, we blocked membrane CD83 on effector CD4^+^ T cells instead, leading to the opposite results.

Subsequently, we tested the direct effect of recombinant sCD83 on a CD4^+^ T cell culture. We failed to show any significant change in CD4^+^ T cell function. Previous reports showed that sCD83 influences T cell activity via induction of tolerogenic DCs (47, 60, 80). The higher expression of sCD83 we observed in lymphocytes from pregnant mice may have a favorable effect on tolerogenic DCs to coordinate innate as well as adaptive mechanisms to support implantation and progression of pregnancy (81).

A deeper understanding of the physiological functions of sCD83 during pregnancy might support the development of therapeutic tools for the treatment of pregnancy- and inflammation-related disorders.

## Ethics Statement

Animal experiments were carried out according to institutional guidelines approved by the Landesamt für Landwirtschaft, Lebensmittelsicherheit und Fischerei Mecklenburg-Vorpommern (LALLF-MV; 7221.3-1-068/13 to DM). The experiments were conducted in conformity with the European Communities Council Directive 86/609/EEC.

## Author Contributions

KP performed experiments, analyzed data, and contributed to the elaboration of the manuscript. JE and DK performed experiments. GR-S designed and performed experiments and contributed to the writing of the manuscript. MZ contributed with reagents, the design of experiments, and the writing of the manuscript. DM conceived and designed the experiments, analyzed data, contributed with reagents, wrote the paper, and supervised the work.

## Conflict of Interest Statement

The authors declare that the research was conducted in the absence of any commercial or financial relationships that could be construed as a potential conflict of interest.
